# Epidural morphine in COVID ARDS patients with high respiratory drive: a structured summary of a study protocol for a randomised controlled trial

**DOI:** 10.1186/s13063-021-05570-5

**Published:** 2021-09-16

**Authors:** Swagata Tripathy, P. Bhaskar Rao

**Affiliations:** AIIMS Bhubaneswar: All India Institute of Medical Sciences - Bhubaneswar, Bhubaneswar, Odisha India

**Keywords:** COVID-19, Randomised controlled trial, Protocol, Respiratory drive, Epidural morphine, Patient self-induced lung injury, Non-invasive ventilation

## Abstract

**Objectives:**

We aim to study the effect of epidural morphine as a means to reduce high respiratory drive in COVID 19 patients on non-invasive ventilation (NIV)—primary end point—and to study its effect on respiratory parameters, subjective patient comfort, rates of endotracheal intubation, duration of mechanical ventilation and mortality.

**Trial design:**

Parallel group, randomised, double blind, single centre placebo control trial. Allocation ratio 1:1, superiority trial

**Participants:**

Trial site and population—COVID ICU patients in the All India Institute of Medical Sciences (AIIMS) Bhubaneswar, Odisha, India

Inclusion and exclusion criteria

Inclusion criteria

Adult patients on NIV with COVID-19

Exclusion criteria

Metabolic acidosis HCO_3_-< 16 and pH < 7.2. Severe hypoxemia warranting cessation of NIV and intubation, non-acceptance of NIV and proven sepsis. Technical difficulty for epidural catheterization, coagulation abnormalities, low respiratory drive and EOL orders.

Sources or methods of recruitment—daily discussion at 8 am of new admissions to COVID ICU on NIV—consenting adult patients with COVID19 on NIV and high respiratory drive; not meeting exclusion criteria will be recruited for the trial and randomised.

**Intervention and comparator:**

Patients of both groups will be turned to a lateral or sitting position (as comfortable), and an injection of local anaesthetic be given at lumbar 2–3/3–4 space. In the intervention group, an epidural catheter will be inserted using aseptic technique and fixed to the skin.

The control group will have a sham catheter fixed exactly like in the intervention group, but not entering the epidural space.

The intervention group will be administered injection morphine sulphate once every 18–24 h into the epidural space. The doses will be escalated daily (5–10 mg), titrated to effect: escalation limited by hypoventilation resulting in respiratory acidosis (pH < 7.2).

The intervention will continue for a minimum of 2 doses and a maximum of 5 doses (96 h) of morphine. It will be stopped if the epidural catheter gets dislodged before the second dose or the patient is weaned off non-invasive ventilation to high flow mask for a continuous period of 24 h or requires endotracheal intubation.

The patient will be followed up till death or 28 days after ICU discharge.

**Main outcomes:**

Primary outcome—diaphragm thickening index fraction (average of minimum 3 readings)

Secondary outcomes—ventilator parameters, sedation and pain scores, subjective comfort and dyspnoea scores, time to intubation, length of stay on NIV and 28-day mortality

Timing of outcome assessment—every 8th hour assessment for 24 h after the last dose of epidural morphine or 120 h whichever is greater

**Randomisation:**

A central random number list will be kept with the study research assistant. She will randomise according to the numbers available in the list using an allocation ratio of 1:1. An opaque sealed envelope concealing the allotted randomisation code will be dispatched to the ICU team.

**Blinding (masking):**

The assessor, patient, nurses and physicians will be blind to group allocation. One member of the team not involved in research will administer the intervention.

**Numbers to be randomised (sample size):**

Twenty-five patients per group; 50 patients total

**Trial status:**

Protocol version 1. Not recruiting yet. Recruitment to begin by 24 July 2021 and end by 31 August 2022

**Trial registration:**

Central Trials Registry India CTRI CTRI/2021/07/035093. Registered on 23 July 2021. Prospectively registered

**Full protocol:**

The full protocol is attached as an additional file, accessible from the Trials website (Additional file 1). In the interest of expediting dissemination of this material, the familiar formatting has been eliminated; this Letter serves as a summary of the key elements of the full protocol

**Supplementary Information:**

The online version contains supplementary material available at 10.1186/s13063-021-05570-5.

## Supplementary Information

**Additional file 1:** Full protocol.

## Data Availability

The data will be available from the author on reasonable request (tripathyswagata@gmail.com).

