# Supplementary material for: Epidural morphine in COVID ARDS patients with high respiratory drive: a structured summary of a study protocol for a randomised controlled trial
Source: Trials. 2021 Sep 16;22:632. doi: 10.1186/s13063-021-05570-5 (PMC8443904; doi:10.1186/s13063-021-05570-5)
Supplement: Supplementary file 1 — Additional file 1: Full protocol. [file 13063_2021_5570_MOESM1_ESM.docx]

**Epidural Morphine in COVID ARDS patients with a high respiratory drive: a double-blind, randomized controlled trial**

**Introduction**

COVID-19 ARDS (CARDS) is associated with a high respiratory drive. (1) If the high respiratory drives are not reduced by oxygen administration and optimal noninvasive support, persistently strong spontaneous inspiratory efforts simultaneously increase tissue stresses and raise pulmonary transvascular pressures, vascular flows, and fluid leakage (i.e., P-SILI). (2) Progressive deterioration of lung function (a VILI vortex) may then rapidly ensue. The adverse effects of high respiratory drive: fatigue, lung strain, and patient self-induced lung injury P –SILI, when patients are left unintubated, are well known. (3)

The only armamentarium available to the clinician at the bedside to reduce abnormally high respiratory drive is to deeply sedate and paralyze the patient and initiate 'lung-protective ventilation. (1) Partial paralysis after intubation has been attempted to reduce high respiratory drives during weaning from ventilation. (4) Literature is replete with studies highlighting the negative consequences of intubation, mechanical ventilation, sedation, and neuromuscular blockers. (5) Infectious complications of intubation are more significant in these patients. (6) The COVID19 ARDS patient requires much higher doses of sedatives than ARDS of other etiology. (7) An association has been noted between deep sedation, coma, and increased mortality among mechanically ventilated COVID 19 patients. (8)

Opioids reduce dyspnea by altering perception, reducing anxiety, and reducing respiratory drive by acting on respiratory centers in the medulla, alleviating the mismatch between the motor drive and pulmonary afferent feedback. (9) Although considered an adverse effect of systemic and central neuraxial opioids, this property of morphine has been utilized to treat patients with both acute and chronically high respiratory drives. (10,11) Epidural morphine is known to reduce respiratory drive at much lower doses than systemic opioids and has the benefit of fewer side effects (nausea, vomiting, pruritus, and constipation) than intrathecal administration. (12,13)

Respiratory drive assessments by Diaphragmatic pressure-time product per breath (PTP_di_) from oesophageal and gastric pressure recordings or electrical activity of the diaphragm (EAdi) are not available readily to the clinician at the bedside. Although airway occlusion pressures (p0.1) have been studied as a surrogate for a high respiratory drive, (14) PTPdi and EAdi correlate well with diaphragmatic thickening fraction index TFdi (15,16), are easily available at the bedside and have been seen to have good repeatability. (17) Values below or beyond 15-30% have been associated with delayed weaning and hyperinflation of the lung.(18,19)

We have previously initiated a feasibility study to look at the effect of epidural morphine to control high respiratory drive in ventilated patients (CTRI/2021/05/033509). Finding encouraging results in decreasing the drive as assessed by TFdi, while maintaining patient alertness and subjective comfort without serious adverse effects, we seek to study its effect in non-invasively ventilated COVID-19 patients.

The aim is to study the effect of epidural morphine to reduce high respiratory drive in COVID 19 patients on NIV (primary endpoint) and to study its effect on ventilator parameters, subjective patient comfort, rates of endotracheal intubation, duration of mechanical ventilation, and mortality.

**Overview of the trial (Fig. 1)**

*Design*

Parallel group, randomized, double-blind, single-center placebo control trial.

**The research question**

Aim To compare the TFDi (primary endpoint), ventilator parameters, subjective comfort, rate of intubation, length of mechanical ventilation, length of ICU stay, and mortality of moderate to severe COVID 19 patients on noninvasive ventilation (NIV), with and without epidural morphine.

*Objectives*

To compare-

Thickening Fraction index of diaphragm TFdi, surrogate for the respiratory drive (primary outcome measure)

P0.1, mechanical ventilation (MV), respiratory rate (RR), Tidal volume (Vt), subjective comfort, the dose of sedatives needed, Visual Assessment of Pain (VAS), Richmond Agitation and Sedation Scores (RASS), time to intubation, length of mechanical ventilation, the incidence of VAP, incidence of sepsis, COVID 19 markers, length of stay in ICU, length of stay in hospital and mortality between the two groups.

**Trial population**

Trial site(s) and population(s)- COVID ICU patients in AIIMS Bhubaneswar

Inclusion and exclusion criteria- Include- Adult patients with COVID 19 pneumonia 18-80 years of age. Requiring NIV to maintain oxygenation.

Exclusion criteria- Metabolic acidosis HCO3-< 16 or pH < 7.2. Severe hypoxemia warranting cessation of NIV and intubation, non-acceptance of NIV. Chronic opioid user. Uncontrolled sepsis, end of life (EOL) orders.

Sources or methods of recruitment- Daily discussion at 8 am of new admissions to COVID ICU on NIV – assessment for high respiratory drive despite Spo2 > 90 and adequate NIV support

Information for participants- PIS will be provided. Written informed consent will be taken for the patient/ kin of all eligible patients.

**Allocation of interventions**

Methods for randomization and stratification- The study research assistant will keep a central random numbers list. She will randomize according to the list available.

Methods for concealment of allocation- An opaque sealed envelope concealing the allotted randomization code will be dispatched to the ICU team.

**The interventions**

A baseline assessment of all outcome measures as appropriate will be made. Normal body temperature, RASS of -1 or 0, and VAS < 4 will be ensured by administering Midazolam ( titrated to effect) or Paracetamol as warranted.

All patients will be turned lateral or sitting position, and an injection of LA be given at L2-3 or L3-4 space. An epidural catheter will be inserted under all asepsis and fixed to the skin in the intervention group. It will be fixed firmly with antimicrobial incise drapes covering at least the lower one-third of the back. For patients who appear fidgety, catheter tunneling may also be used.

The control group will have a sham catheter fixed precisely like the intervention group but not entering the epidural space. The fixation and dressing will be similar to the intervention group.

The intervention group will be administered Injection Morphine Sulphate once every 18-24 hours into the epidural space. The doses will be escalated daily (5-10mg), titrated to the effect: escalation limited by hypoventilation resulting in respiratory acidosis (pH <7.2).

The intervention will continue for a minimum of 2 doses and a maximum of 4 doses (96 hours) of morphine. It will be stopped at 120 hours, or if: the epidural catheter gets dislodged before the second dose, the patient is weaned off noninvasive ventilation to high flow mask for a continuous period of 12 hours or requires endotracheal intubation.

The patient will be followed up till death or 28 days after ICU discharge.

**Outcome assessment**

*Outcome measures*

TFdi, p0.1, MV, RR, Vt (average of 3 readings, ^eighth^ hourly )

Arterial blood gas and associated values-Pa o2, PaCO2, pH, HCO3, p/F ratio (at least once a day)

Spo2, Fio2, ROX index, Subjective comfort and dyspnea scores – 8^th^ Hourly

VAS, RASS, Weaning (hours off NIV on high flow mask or face mask)- (mean values over 24 hours)

Intubation- Yes/ No: Days after randomization

Length of stay in ICU

Outcome at 28 days of admission

Incidence of excessive respiratory depression (PaCO2> 45 and pH < 7.2)

Incidence of difficult/ failed epidural insertion, catheter pullouts, and complications such as bleeding/ infection/ hematoma will be documented.

Timing of outcome assessment- - Every 8^th^-hour assessment / daily / one time as detailed above

Blinding- The assessor, patient, nurses, physicians, and treating team will be blind to group allocation. One member of the team not involved in research will administer the drug.

**Post-recruitment retention strategies**

Participant retention- Follow-up will be till stay in the hospital or 28 days, whichever is longer.

**Safety monitoring and adverse events**

Data and safety monitoring- Adverse effects will be documented and reported to the IEC.

**Data collection and management**

Data collection- Routine data will be extracted from the ICU records; Study-specific outcome data will be collected every eigh^th^-hour Data collectors will be distinct from the persons administering the epidural morphine/ vs. placebo- (in this case, air)

Photographic evidence will be kept of ventilator parameters, USG of the diaphragm, and subjective comfort and dyspnea scores at each data collection point and sent from inside the COVID ICU to the investigator who is not involved in the patient's clinical care and unaware of group allocation via Whatsapp.

Data entry- Will be done from the photographs and paper Case Report Forms (CRFs) into an Excel sheet by a researcher blinded to the group allocation.

For each time point, representative photographs (de-identified) will be kept for possible crosschecking and data validation as required.

**Sample size**

Sample size justification A pilot study showed the mean TFDi in COVID 19 patients on PEEP 5 to be 45%(SD 15); To decrease mean fractional thickening of the diaphragm index by 30% after the dose of morphine with allocation in a 1:1 ratio, 21 patients will be needed in each group. Accounting for dropouts/ loss of data, 25 patients will be recruited in each limb.

*Statistical analysis*

Interim analyses are planned at 50% of sample recruitment, without unblinding to ensure that subjects are not being exposed to an unacceptable level of risk, whether the trial can be stopped early, check some of the assumptions that went into the original design and sample-size calculations of the trial (like within-group variability, recruitment rates, base event rates). The alpha spending strategy used will be one-fifth of the available 5 percent alpha at the interim analysis. i.e., the interim analysis p-value must be < 0.01 to stop the trial early and claim efficacy. Spend the remaining four-fifths of the 5 percent alpha at the end, or the end analysis p-value must be < 0.04 to claim efficacy. The two groups shall be compared for the mean TFdi (overall and 24^th^ hrly) by unpaired t-tests. Within-group comparison of TFdi shall be made (overall and 24^th^ hrly) by Repeated measures analyses.

Secondary outcomes- Intubation, Mortality, duration of mechanical ventilation, ICU stay, Subjective scores, Demographics, and severity of illness shall be compared by chi-square, t-tests, or nonparametric tests as suitable.

**Ethical aspects**

Written informed consent shall be taken from the participant or the next of kin.

**Trial management**

Prospective registration in Central Trials Registry India (CTRI) **CTRI/2021/07/035093** on Jul 23, 2021.

*Trial management- By PI*

*Local coordination- By PI*

*Research governance and good clinical practice- shall be followed.*

**Economic evaluation- NA**

**Consumer involvement NA**

**Reporting, Dissemination, and Notification of results**

*Publication policy- Results will be collated and manuscript prepared for submission to a PubMed indexed journal.*

*Disseminating the results- Results will be disseminated in academic meets, social media as per rules.*

**Detailed methodology, with definitions**

**PATIENTS**

- Inclusion: 18 -80 years of age; met all 4 of the following criteria
  - **RR** > 25 breaths per minute; **PaO2/FiO2** < 200mmHg while the patient was breathing oxygen at a flow rate of ≥ 10 liters per minute for at least 15 minutes;
  - **On NIV PSV6 PEEP 5** with < 30% leak**, Minute ventilation > IBW x10mlx25/min** (e.g., for 50 kg individual = 12.5 lpm)
  - **Rule out pain, fever, discomfort**

If any present:

Manage with (when in doubt, all can be given together-)

Injection PCM 1gm- stat for pain/discomfort and /or fever,

Injection Midazolam 1 mg iv stat for anxiety,

Midazolam to be followed by Infusion Dexmedetomidine 400 mcg in 50 ml at 3-5 ml per hour, titrated to blood pressure ( MAP >65 mmHg or and effect.

*And* active patient counseling to comfort and reassure)

**Reassess Minute volume after 30 minutes.**

- Exclusion: PaCO2 ≥ 45 mmHg *and* pH < 7.2; Compensated respiratory acidosis was accepted up to a pH 7.2 according to standard ARDS lung-protective ventilation guidelines *if GCS >12*

Compensated metabolic acidosis with HCO3 < 16mmol/l with sepsis/ acute or chronic renal failure,

Hemodynamic instability (MAP< 65 mmHg) *and* need of increasing doses of vasopressors in last 6 hrs.; GCS <12;

Contraindication to noninvasive ventilation;

Urgent need for intubation (evaluated by the clinician in charge)

Refusal to participate or participate in another study.

Do-not-intubate decision

Epidural catheter block/ dislodged/ less than two doses of the drug could be administered.

**NIV INITIATION**

**Determine if the patient is a candidate for NPPV**

1 Conscious, maintaining airway, and breathing spontaneously for several minutes in case of mask displacement.

Also, meet at least 1 of the following criteria: >

- Respiratory distress- moderate to severe dyspnea and respiratory rate > 25 with accessory muscle use and/or paradoxical breathing and unable to speak a complete sentence with PaO2/FiO2 < 200 or SpO2< 92% on high flow mask with >15lpm oxygen flows

Select and install patient interface and breathing circuit

**Manage patient anxiety**

- Explain the goal of noninvasive ventilation
- Prearrange patient communication
- Start with low-pressure settings and hold the mask to the patient's face before tightening the strap.
- Let the patient remove the mask for a short time to speak or drink.

**Set the controls**

Adjust the settings as needed to optimize synchronization, optimize breath volume and/or PCO2, minimize fatigue of accessory muscles, relieve dyspnea, and reduce respiratory rate—the frequency goal to be ≤ 25 b/min. Titrate ventilation and oxygenation. Adjust settings as patient's condition and leak change, and adjust the alarms appropriately.

**Optimize patient comfort**

> Make sure the mask fits properly. Check repeatedly that the mask is comfortably positioned. Maintain an acceptable leak tolerance. A small leak is well compensated and should be present to prevent pressure necrosis avoid leaks >30%

**Minimize adverse effects**

Pressure sores > Check whether the mask is the right size and is appropriately positioned. Use wound care dressing, adequate upper airway humidification, heated humidifier, saline nasal spray, an oilless salve to the affected areas as needed. Excessive leakage > Adjust the interface, adjust the straps, and/or change to another type of mask.

**EPIDURAL**

Baseline PT/ INR and platelet levels should be within an acceptable range to allow epidural insertion. Insertion catheter between 9 to 2 pm on the given day per the protocolized LMWH given at 7 pm on the units: or at least 4 hrs before the next dose of UFH and 12 hours after the last dose.

**Remove** the **catheter** a minimum of 4 -6 hours after a dose of UFH. Administer the next dose of UFH no sooner than 2 hours after **catheter removal**. Once-daily low-molecular-weight heparin (LMWH).

Daily site inspection (antimicrobial incise drapes transparent dressing) is done each time the drug/ sham injection is made. The injection port is kept well covered and sealed and brought to the side near the flank/ shoulder. The epidural catheters (True or Sham) will be in situ for a minimum of 2 doses or 48 hours, whichever is more, and a maximum of 4days (96 hours of catheter and 120 hours of observation).

All asepsis will be maintained for the control group as in the Intervention group, a Local injection anesthetic will be given at the L2-L3/ L3-L4 site, and the catheter will be taped tightly to the skin and dressing done to match the intervention group.

**WEANING NIV SETTINGS**

The clinical and physiological parameters used in this setting include – arterial pH ≥ 7.35, oxygen hemoglobin saturation (SpO_2_) > 88% on FiO_2_ ≤ 60%, respiratory rate ≤ 25 / min, heart rate ≤ 120 / min, systolic blood pressure ≥ 90 mmHg, and no signs of respiratory distress like agitation, diaphoresis or anxiety.

Patients satisfying these criteria may be weaned by any method as per clinician discretion- step reduction of the duration of NIV use, stepwise reduction in pressure support, and immediate intermittent withdrawal of NIV.

**CRITERIA FOR ENDOTRACHEAL INTUBATION**

The pre-determined criteria for endotracheal intubation and mechanical ventilation (MV) to avoid delayed intubation:

1. Signs of persisting or worsening respiratory failure:

- RR > 40 cycles/min,
- Signs of respiratory-muscle fatigue (no improvement in subjective scores for > 12 hours)
- Copious tracheal secretions,
- SpO2 below 85 % for more than 5 min without technical dysfunction at Fio2 100% and best-tolerated PEEP
- Intolerance to NIV

or one of the following

1. Hemodynamic instability is defined by an SBP below 90 mmHg, MBP below 65 mmHg, or requirement for increasing vasopressor doses to maintain the same for 2 hours.
2. Deterioration of neurologic status with a Glasgow coma scale below 12 points.

For all patients, a trial of HFNO or Nonrebreather oxygen will be allowed according to the physician's preference in patients with signs of persisting or worsening respiratory failure (to rule out patient discomfort with NIV) and no other organ dysfunction before performing endotracheal intubation and invasive ventilation.

**DATA COLLECTION**

The following data will be recorded on admission:

**Demographic & ICU admission data;**

Age, gender, Carlson's comorbidity Score, APACHE-II, Sepsis-related Organ Failure Assessment (SOFA) score, (scores range from 0 to 24, with higher scores indicating more severe organ failure), COVID markers on admission, baseline assessment, and final assessment ( Serum ferritin, IL6, CRP where available)

The number of lung quadrants involved in chest X-rays at admission or CT severity score if available.

**Physiological parameters** including respiratory rate, systolic blood pressure, and heart rate, and arterial-blood gases will be recorded at inclusion, between 12 and 24 hours after randomization and minimum once every 24 hours after that.

**Respiratory Drive and related parameters**

Once at baseline and once every 8 hours after that, the following will be assessed till the epidural catheter is in situ, or five days whichever is greater.

The researcher will do the baseline assessment, be aware of the group allocation, and insert the epidural catheter (true or sham). She/ He will mark the site of best visualization for USG probe, ensure best fit NIV interface and assess baseline subjective comfort scores.

After that, all assessments will be done by a fixed set of postgraduate ICU trainees, all trained in TFDi assessment but blinded to group allocation.

Ultrasound-guided TFDi- This procedure will be performed as described earlier.(17,19)

The patient will be supine with head-end elevated to 30 %. The right hand will be abducted and kept near the head. The diaphragm will be located by placing the transducer in the intercostal space above the right 8^th^- 11^th^ intercostal space, in the anterior- axillary line, directing the ultrasound beam perpendicular to the diaphragm. The inferior border of the costophrenic sinus will be identified at end-inspiration as the zone of transition from the lung sliding shadow to the visualization of the diaphragm and liver. The zone of apposition will be noted.

The diaphragm thickness will be recorded in time motion (TM) mode.

The sweep speed will be adjusted to obtain a minimum of three to 8 cycles on the same image.

Several diaphragm images (outlined by the two clear, bright parallel lines of the pleural and peritoneal membranes) will be located: the image will be discarded if the two bright lines are not visible. The least and maximum thickness as on eyeballing will be located and a picture stored for subsequent quantitative analysis and crosschecking by a blinded assessor.

The position of the first (baseline) USG assessment will be marked on the skin to allow all subsequent recordings from the same position.

A note will be made of the ongoing sedation dosages and ventilator settings during the measurement of TFdi (settings will be kept the minimum required for patient comfort – but will not be changed/ dictated for purposes of the study)

Airway occlusion pressures (p0.1), Respiratory rate/ min, Tidal volume (Vt) & Minute Ventilation (MV) as displayed on the ventilator at the ongoing minimal support for best patient comfort and oxygenation: observed over 1 minute, an average of 3 highest readings (ensuring a mask leak < 30%) All patients will be on Hamilton C1 ventilators.

**Epidural Catheter**

Ease of insertion and complications during catheter insertion and removal- Bleeding/ difficult or impossible at the hands of experts/ hematoma/ catheter break/ accidental removal/ blockage/ other.

**Analgesia, Sedation, Fever**

VAS and RASS scores are charted four^th^ hourly in the ICU- a daily mean score will be collected from the daily data sheets of the patients.

Any temperature > 101 F will be noted as the number of episodes per day.

**NIV weanability**

Once a day, daily data will be collected as- weaned fully from NIV/ weaning from NIV ongoing/ same as yesterday/ not wean-able/ respiratory support increased/intubated)

Hours off NIV will be recorded as – 0/ <12/ 12-18/ >18 and off NIV

**Respiratory patient-discomfort and dyspnea**

Discomfort- Unmarked 100 mm visual analogic scale from "no discomfort" to "maximal imaginable discomfort,"

Dyspnea- Likert scale model indicating marked improvement (+2), slight improvement (+1), no change (0), slight deterioration (-1) and marked deterioration (-2).

**Intubation and mortality rates,**

Duration of NIV and invasive mechanical ventilation

Cause for conversion to IMV- Progressive respiratory failure/ New Sepsis/ Neurologic dysfunction/ Hemodynamic instability

The length of ICU-stay,

The organ dysfunctions and adverse events, including cardiac dysrhythmia, septic shock, and cardiac arrest, will be recorded until the ICU stay.

Mortality will be followed today 28.

**Sample size**

Sample size justification A pilot study showed the mean TFDi in COVID 19 patients on PEEP 5 and clinically optimal pressure support to be 45% (SD 15); To decrease this by 30% at 36 hours after the dose of morphine with allocation in a 1:1 ratio, 21 patients will be needed in each group.

Compliance and missing data- All analyses will be based on the groups as randomly allocated: an intention-to-treat analysis. A per-protocol analysis will also be done. The statistical significance will be taken as the 5% level for the top comparisons and the subsidiary comparisons, the 1% level. Patients with a P/F ratio > 150 at inclusion will be compared to those with lower values.

Data Storage- Because of the unique challenges of repeated data capture in COVID ICU, all repetitive data will be captured photographically and sent to a central data repository online to be categorized and stored by patient name and time of data capture (once every 8 hours), making in 14-time points of data capture for each patient. Data storage will be by blinded personnel.

**References**

1. Marini JJ, Gattinoni L. Management of COVID-19 Respiratory Distress. Vol. 323, JAMA - Journal of the American Medical Association. American Medical Association; 2020. p. 2329–30.

2. Spinelli E, Mauri T, Beitler JR, Pesenti A, Brodie D. Respiratory drive in the acute respiratory distress syndrome: pathophysiology, monitoring, and therapeutic interventions. Intensive Care Med. 2020;46(4):606–18.

3. Esnault P, Cardinale M, Hraiech S, Goutorbe P, Baumstarck K, Prud'homme E, et al. High Respiratory Drive, and Excessive Respiratory Efforts Predict Relapse of Respiratory Failure in Critically Ill Patients with COVID-19. Am J Respir Crit Care Med. 2020;1–17.

4. Doorduin J, Nollet JL, Roesthuis LH, Van Hees HWH, Brochard LJ, Sinderby CA, et al. Partial neuromuscular blockade during partial ventilatory support in sedated patients with high tidal volumes. Am J Respir Crit Care Med. 2017;195(8):1033–42.

5. Tobin MJ, Laghi F, Jubran A. Caution about early intubation and mechanical ventilation in COVID-19. Ann Intensive Care. 2020;10(1):4–6.

6. Giacobbe DR, Battaglini D, Enrile EM, Dentone C, Vena A, Robba C, et al. Incidence and Prognosis of Ventilator-Associated Pneumonia in Critically Ill Patients with COVID-19: A Multicenter Study. J Clin Med 2021, Vol 10, Page 555. 2021 Feb 3;10(4):555.

7. Flinspach AN, Booke H, Zacharowski K, Balaban Ü, Herrmann E, Adam EH. High sedation needs of critically ill COVID-19 ARDS patients—A monocentric observational study. Lazzeri C, editor. PLoS One. 2021 Jul 27;16(7):e0253778.

8. Wongtangman K, Santer P, Wachtendorf LJ, Azimaraghi O, Baedorf Kassis E, Teja B, et al. Association of Sedation, Coma, and In-Hospital Mortality in Mechanically Ventilated Patients With Coronavirus Disease 2019–Related Acute Respiratory Distress Syndrome. Crit Care Med. 2021; Publish Ah:1–11.

9. Mahler DA. Opioids for refractory dyspnea. https://doi.org/101586/ers135. 2014 Apr;7(2):123–35.

10. Johnson MJ, Bland JM, Oxberry SG, Abernethy AP, Currow DC. Opioids for chronic refractory breathlessness: Patient predictors of beneficial response. Eur Respir J. 2013 Sep 1;42(3):758–66.

11. Soffler MI, Rose A, Hayes MM, Banzett R, Schwartzstein RM. Treatment of Acute Dyspnea with Morphine to Avert Respiratory Failure. http://dx.doi.org/101513/AnnalsATS201611-922CC. 2017 Mar 31;14(4):584–8.

12. Etches RC, Sandler AN, Daley MD. Respiratory depression and spinal opioids. Can J Anaesth. 1989;36(2):165–85.

13. MA C. Side effects of intrathecal and epidural opioids. Can J Anaesth. 1995 Oct;42(10):891–903.

14. Spinelli E, Mauri T, Beitler JR, Pesenti A, Brodie D. Respiratory drive in the acute respiratory distress syndrome: pathophysiology, monitoring, and therapeutic interventions. Intensive Care Med. 2020;46:606–18.

15. Vivier E, Roche-Campo F, Brochard L, Mekontso Dessap A. Determinants of diaphragm thickening fraction during mechanical ventilation: an ancillary study of a randomized trial. Eur Respir J. 2017;50(3).

16. Correlation Between Diaphragm Thickening Fraction and Electrical Activity | C76. CHEST WALL, RESPIRATORY MUSCLES: NEURAL AND VENTILATORY CONTROL [Internet]. [cited 2021 Aug 3]. Available from: https://www.atsjournals.org/doi/abs/10.1164/ajrccm-conference.2018.197.1_MeetingAbstracts.A5870

17. E V, A MD, S D, F V, A L, AW T, et al. Diaphragm ultrasonography to estimate the work of breathing during noninvasive ventilation. Intensive Care Med. 2012 May;38(5):796–803.

18. Goligher EC, Fan E, Herridge MS, Murray A, Vorona S, Brace D, et al. Evolution of Diaphragm Thickness during Mechanical Ventilation. Impact of Inspiratory Effort. https://doi.org/101164/rccm201503-0620OC. 2015 Oct 30;192(9):1080–8.

19. Tuinman PR, Jonkman AH, Dres M, Shi Z-H, Goligher EC, Goffi A, et al. Respiratory muscle ultrasonography: methodology, basic and advanced principles and clinical applications in ICU and ED patients—a narrative review. Intensive Care Med 2020 464. 2020 Jan 14;46(4):594–605.

Fig 1. Study flow

**
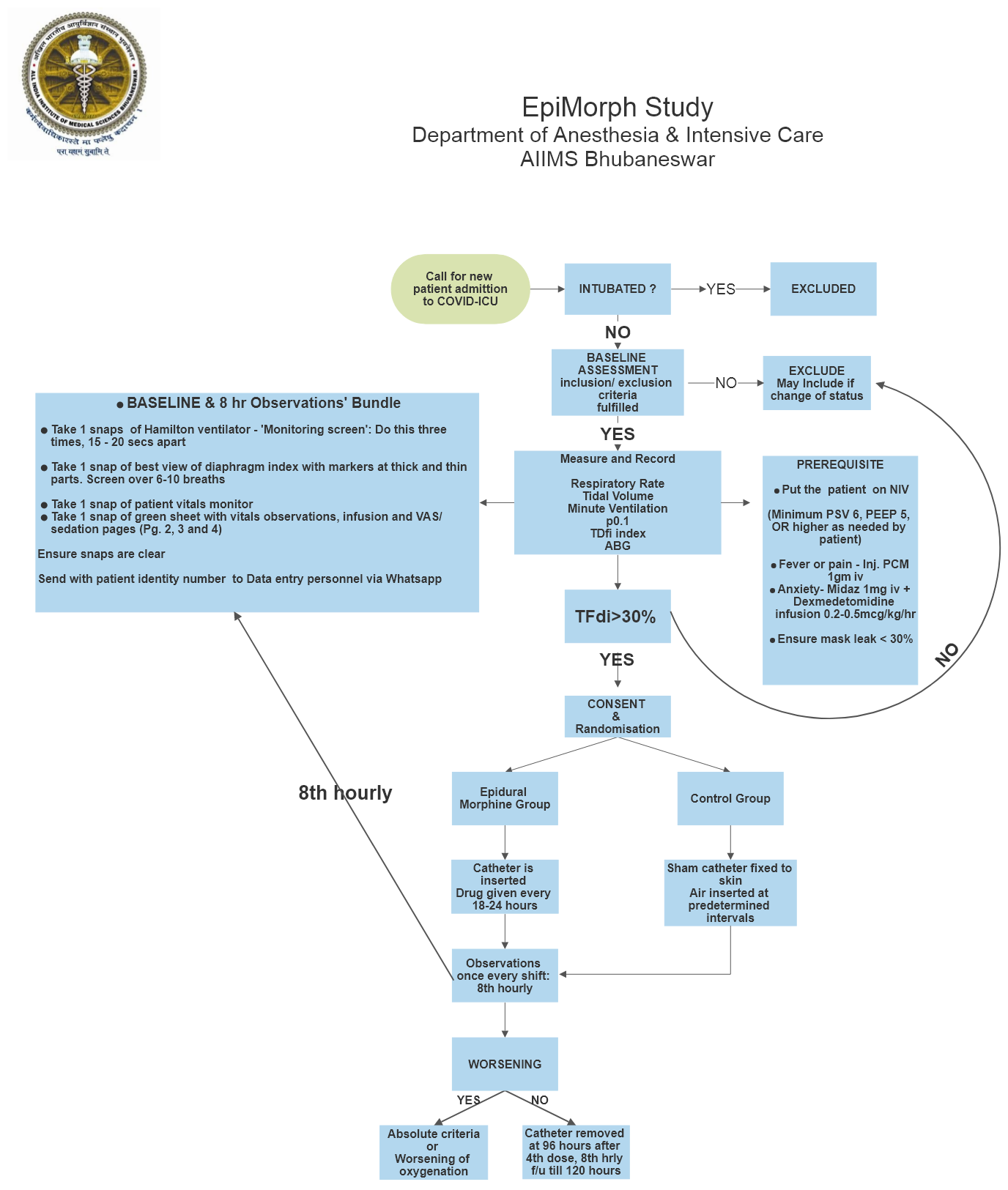
**
